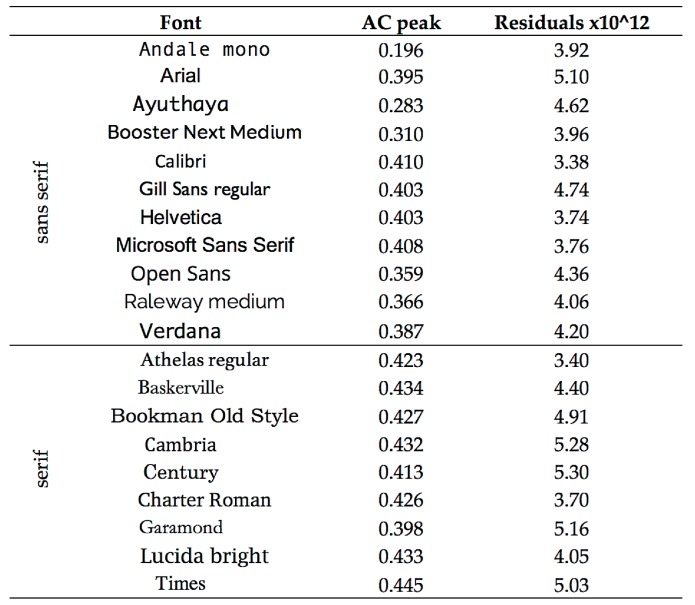# The Influence of Typography on Algorithms that Predict the Speed and Comfort of Reading

**DOI:** 10.3390/vision4010018

**Published:** 2020-03-12

**Authors:** Arnold Wilkins, Katie Smith, Olivier Penacchio

**Affiliations:** 1 Department of Psychology, University of Essex, Wivenhoe Park, Essex, Colchester CO4 3SQ, UK; kasmith2005@hotmail.co.uk; 2 School of Psychology and Neuroscience, University of St. Andrews, St Mary’s Quad, South Street St Andrews, Fife KY16 9JP, UK; op5@st-andrews.ac.uk

**Keywords:** font, spatial periodicity, discomfort, reading speed, autocorrelation, Fourier amplitude spectrum

## Abstract

1. The speed with which text can be read is determined in part by the spatial regularity and similarity of vertical letter strokes as assessed by the height of the first peak in the horizontal autocorrelation of the text. The height of this peak was determined for two passages in 20 fonts. The peak was unaffected by the size of the text or its content but was influenced by the font design. Sans serif fonts usually had a lower peak than serif fonts because the presence of serifs usually (but not invariably) resulted in a more even spacing of letter strokes. There were small effects of justification and font-dependent effects of font expansion and compression. 2. The visual comfort of images can be estimated from the extent to which the Fourier amplitude spectrum conforms to 1/f. Students were asked to adjust iBooks to obtain their preferred settings of font and layout. The preference was predicted by the extent to which the Fourier amplitude spectrum approximated 1/f, which in turn was jointly affected by the design of the font, its weight and the ratio of x-height to line separation. Two algorithms based on the autocorrelation and Fourier transformation of text can be usefully applied to any orthography to estimate likely speed and comfort of reading.

## 1. Introduction

There have been many studies of the effects of typographic variables on reading [1,2] including the effects of letter size (*x-height*) [3], line spacing (*leading*) [4] and typeface (e.g., *serif* vs. *sans serif* [5]. Text is a complex stimulus in which the effects of typographic variables interact together and with other variables such as familiarity [6] to determine the speed and comfort of reading. In this paper, it is shown that despite this complexity, simple algorithms that measure the shape of text are able to predict both reading speed and choice of font. They do so by registering the extent to which the spatial periodicity of text interferes with vision, and may have relevance for both normal and low-vision reading. One algorithm measures the periodicity from letter strokes, whereas the other measures aspects of the periodicity from lines of text.

## 2. Horizontal Spatial Periodicity

**Figure 1:**
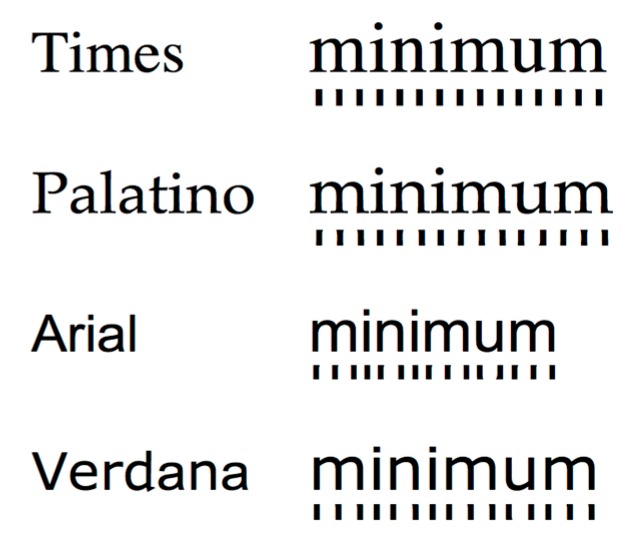
The horizontal periodicity in the letter strokes for the word minimum printed in Times, Palatino, Arial and Verdana. The font size has been chosen to equate the x-height. The pattern beneath each word is that formed by the central part of the letter strokes.

Weaver [7] reported the case of a lady who experienced seizures when reading. She found that the seizures occurred only when she was reading material printed in Times or Palatino, and not when reading the same material printed in Arial or Verdana. Her observations were confirmed electrophysiologically. Times and Palatino are fonts with serifs, whereas Arial and Verdana are sans serif fonts. Serifs have little effect on reading [5]. However, the fonts differ not only with respect to serifs but in the periodicity of the letter strokes, as illustrated in Figure 1. In Arial and Verdana, the space between the strokes within a letter is greater than that between letters, whereas in Times and Palatino, the letter strokes are evenly spaced, giving the typeface a spatial regularity referred to by typographers as *rhythm*.

The rhythm of a typeface can be assessed using horizontal autocorrelation, the correlation of an image with a second version of itself, displaced horizontally by a small amount (lag). The mathematical technique can be understood by imagining text printed on the transparency of an overhead projector. When two identical transparencies are superimposed and in register, the light transmitted through them is at its maximum (the correlation is 1.0). When the top transparency is moved horizontally across the lower one, the lag increases and the transmitted light decreases (the correlation decreases). It reaches a minimum when a majority of letter strokes on the top transparency are in the spaces between letters on the lower transparency. As the top transparency is moved further, and the lag increases further, the transmitted light increases (and the correlation increases), reaching a maximum when the majority of letter strokes on one transparency lie on the neighbouring letter strokes on the other. 

The height of the first peak in the horizontal autocorrelation is therefore a measure of the spatial periodicity of the typeface, dependent on both the shape and spacing of neighbouring letter strokes. Wilkins et al. [8] showed that the height of the first peak in the horizontal autocorrelation of a word determined how striped in appearance the word was judged to be. More importantly, they showed that the height of the peak predicted the speed with which the word could be read aloud. Words with high peak were read about 10% more slowly than those with low. The peak also predicted the speed of silent visual search through a paragraph of text. Reducing the autocorrelation by compressing words near the middle and expanding them at the edges (leaving their length unchanged) increased reading speed, even though readers preferred to read an undistorted version of the text [8]. One of the reasons for the effects of spatial periodicity on reading speed concerns the ways in which the eyes move across text when reading.

During a rapid eye movement (saccade), one eye generally leads and the other follows, resulting in a misalignment of the eyes that requires correction when the eyes come to rest [9]. Jainta, Jaschinski, and Wilkins [10] measured saccades and vergence movements when their participants read German sentences. When the words had a high first peak in the horizontal autocorrelation, the eyes rested on each word for longer during the vergence eye movements that corrected the misalignment (vergence error). The realignment took longer with a spatially periodic word because the alignment was then more precise.

Little is known about the effect of typographic variables on the size of the horizontal autocorrelation, even in the two studies in which the spatial periodicity has been shown to affect reading. The first of the present studies therefore measured the effect of typographic variables on the autocorrelation.

## 3. Study 1

Study 1 explored the way in which the first peak in the horizontal autocorrelation of text varies with font and layout. Twenty common fonts were selected, shown in the first column of Table 1.

### 3.1. Comparison of 20 Fonts

#### 3.1.1. Procedure

A passage from “Small House at Allington” by Anthony Trollop and a passage from “Middlemarch” by George Eliot were generated using Microsoft Word for Mac 2011 version 14.6.8. They were printed to the 15 inch Retina screen of an Apple Macbook Pro running OSX 10.11.6. The passage was prepared in each of the 20 fonts listed in Table 1, nominal 10 point in size, with default letter spacing, and with a ragged right margin and an interlinear spacing of 1.15 points. The page was 20.5 cm in width and 28.5 cm in height and was saved with a resolution of 5.67 pixels per mm. The horizontal autocorrelation of a central section of the page 512 × 512 pixels in size was obtained using Matlab with iteration of the *corr2d* function.

#### 3.1.2. Results

**Figure 2:**
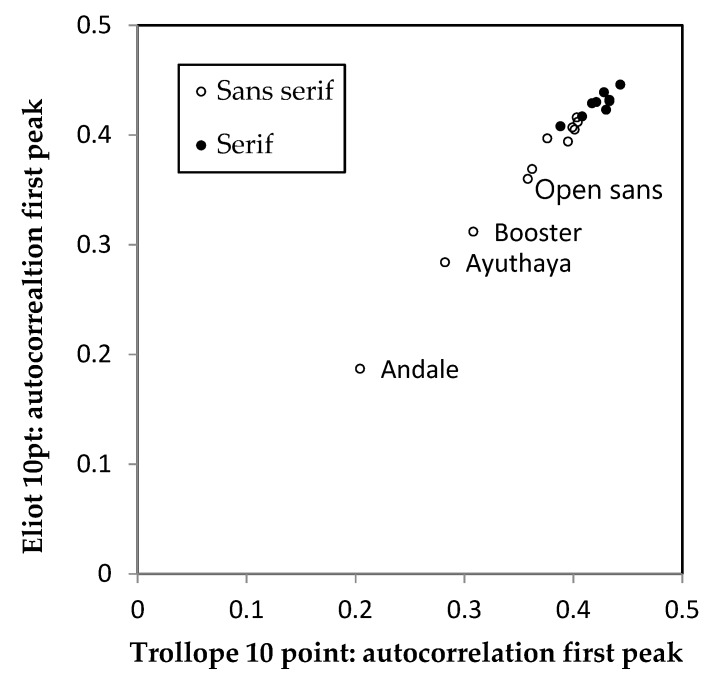
The first peak in the horizontal autocorrelation of two pages of text, one containing text from Eliot and the other from Trollope. Both samples were printed in one of 20 fonts. The fonts with low first peak in the autocorrelation have been identified.

Figure 2 shows a plot of the first peak in the horizontal autocorrelation for the 20 fonts. As can be seen, the peak is very similar for the two passages of text but differs for each font. The absolute value of the difference in the autocorrelation for the two text samples averaged only 0.0092 (SD 0.0064), demonstrating that the method shows reliable differences attributable to the font design, and not the content of the text. The mean value for each font is shown in the second column of Table 1.

As can be seen from Table 1, the font with lowest autocorrelation was a “typewriter” font in which each letter had the same width. Of the proportional fonts, the two with lowest autocorrelations were Open Sans and Raleway medium. Arial and Verdana had autocorrelations less than 0.4. Although these are all sans serif fonts, the remaining cluster of fonts with autocorrelations greater than 0.4 included many fonts with serifs, but also Gill sans. The difference in the first peak in the horizontal autocorrelation was significantly greater for the fonts with serifs than those without t(18) = 3.45, *p* = 0.003.

#### 3.1.3. Discussion

There were differences in the horizontal autocorrelation of a page of text that were due almost entirely to the font (typeface) and not the content of the text. Evidently, a single page of text provides for averaging sufficient to remove most of the variability from content.

### 3.2. Serifs Versus Rhythm

**Figure 3:**
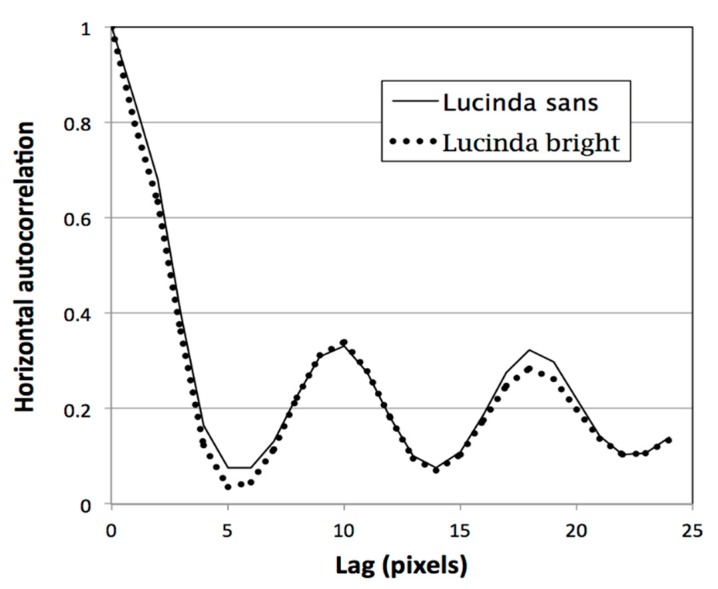
The horizontal autocorrelation of two identical passages of text from Trollope, one in Lucia sans and the other Lucinda bright.

The difference in the first peak of the horizontal autocorrelation between the serif and sans serif fonts was not due to the serifs themselves, but the effect of the serifs on the *rhythm* of the typeface, evident in Figure 1. This was demonstrated by a subsequent comparison of Lucida bright and Lucida sans. The letter shapes and spacings are similar in the Lucida typefaces although Lucida bright contains serifs. Two identical passages from Trollope were prepared, one in Lucida bright and one in Lucida sans, each 12 point. The tracking (letter spacing) was identical. The autocorrelations of the two typefaces are shown in Figure 3 and are very similar. The first peaks in the autocorrelations were 0.323 and 0.284 respectively. Evidently, the serifs per se did not contribute much to the first peak in the horizontal autocorrelation. Perea [11] compared serif and sans serif versions of Lucida and found no effect of serifs on mean fixation duration and only minor effects on the number of progressive saccades.

### 3.3. Equating Empty Space

Although all the fonts had the same nominal point size, they differed in width and in the height of the central body of the letters (x-height). The x-height of Times nominal 14 pt was similar to that of Google Open Sans nominal 12 pt, for example. Although the line spacing was nominally 1.15 pt, it differed from one font to the next, partly to maintain a similar ratio of x-height to line space. Other things being equal, the larger the area of empty white page, the greater the autocorrelation. The variation between fonts therefore depended in part on this variation and not on the spatial periodicity between letters. Further, the smaller the size of the letters, the fewer the pixels used to create the letter form, and the greater the degradation in shape.

#### 3.3.1. Procedure

In order to compare the autocorrelation of different fonts independently of these factors, the text from Trollope was created in 28 point, and the size normalised to give an x-height of 27 pixels for all fonts, using the imresize function in Matlab (bicubic interpolation), cropping the width of the fragment at 1168 pixels.

#### 3.3.2. Results

**Figure 4:**
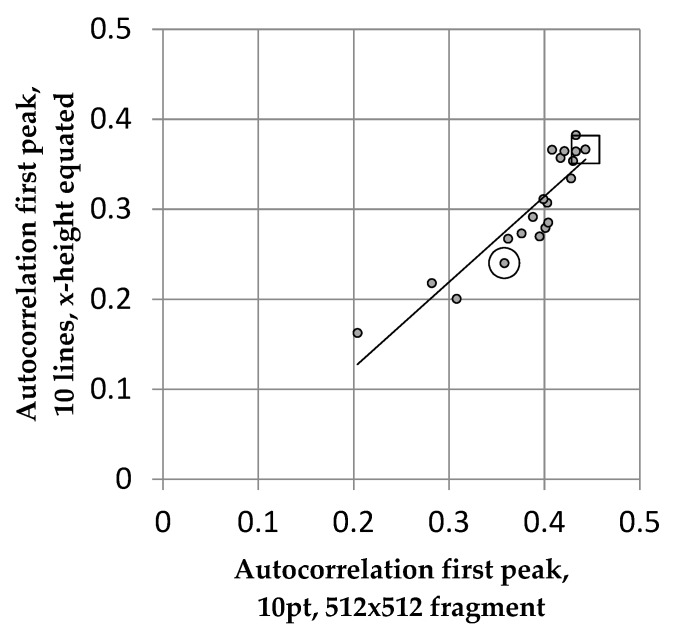
Correlation between text from Trollope 10 pt with 1.15 pt interline spacing and the same passage normalised for x-height, for the 20 fonts listed in Table 1. The points for Open Sans and Times are surrounded by a circle and a square respectively.

Figure 4 shows the relationship between the autocorrelation obtained using this method and that originally obtained for the 10 pt font. The correlation is 0.91.

#### 3.3.3. Discussion

Evidently, the first peak in the horizontal autocorrelation is largely independent of any image changes associated with scale and spacing, at least over the range of scale examined. Google Open Sans is surrounded by a circle and Times by a square in Figure 4. Note that Google Open Sans is consistently a font with low autocorrelation, and Times one with high autocorrelation.

### 3.4. Horizontal Spacing

#### 3.4.1. Font Compression and Expansion

The spacing of letters may be expected to affect the lag at which the autocorrelation reaches its peak, but the effect of expansion on the height of the first peak in the autocorrelation depends on the intra and interletter space and is not easily predicted. Wilkins et al. [8] showed that for Times the autocorrelation is at its peak when the letter spacing is default: the peak decreases with both expansion and compression. By contrast, Verdana showed comparatively little change with letter spacing. The effects of interword and interletter spacing on reading are known to interact with font characteristics [12].

#### 3.4.2. Justification

The word processor used in the previous studies achieved right justification by varying the space between but not within words, and the average spacing between words was increased when the text was justified. The passage by Trollop was printed using Microsoft Word in one of four fonts (Times, Century, Verdana and Open Sans). Each was printed in one of three nominal sizes (10 pt, 12 pt, 14 pt) with both right justification and ragged right margins—a total of 24 samples. 

The autocorrelation of the 24 samples was obtained using an algorithm that analysed 10 lines without interline spacing. The effect of font size was inconsistent, but there was a small but consistent effect of justification: it increased the first peak in the autocorrelation for all four fonts, although only by an average of 3.3%. The average spacing between words increased when the text was justified and the extra blank white page increased the autocorrelation.

## 4. Discussion of Study 1

The first peak in the horizontal autocorrelation of words has been shown to influence the speed with which they can be read both in a list [8] and as connected meaningful sentences [10]. The peak is high when vertical strokes of letters resemble each other and are evenly spaced, and words are then read more slowly [8,10]. Serif fonts generally have a higher peak, not because of the serifs themselves but because of their effect on the *rhythm* of the typeface. Other aspects of text spacing and layout have a comparatively small effect. Although the peak has been shown to influence reading speed, familiarity with font design is, however, also likely to influence speed of reading [6], and this may have a larger effect. 

## 5. Vertical Spatial Periodicity

Certain periodic patterns, particularly stripes, can be uncomfortable to look at. They can induce perceptual illusions of movement, shape and colour [13]. The patterns responsible for discomfort and illusions have characteristics similar to those of patterns that induce electroencephalographic abnormalities, or seizures, in patients with photosensitive epilepsy, suggesting a neurological rather than optical basis for the visual effects [14]. There are large individual differences in susceptibility to the illusions and associated discomfort and these differences are related to a person’s history of headaches. There are several convergent lines of evidence that the visual cortex is hyperexcitable in migraine headache. It is therefore parsimonious to explain the individual differences in susceptibility to the visual effects of stripes in terms of individual differences in cortical excitability [15]. The successive lines of text resemble a pattern with spatial frequency, luminance contrast, and duty cycle (the proportion of background luminance in the pattern) sufficient to induce discomfort and even seizures [16,17]. This can best be understood using Fourier analysis.

**Figure 5:**
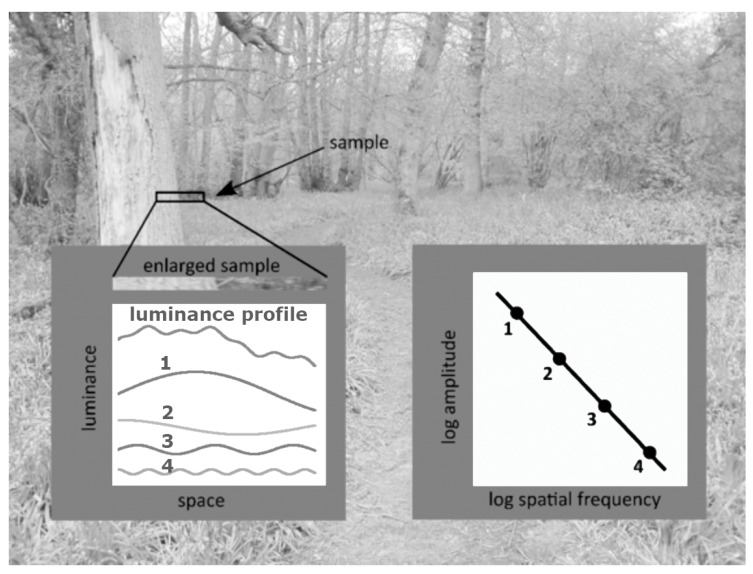
Illustration of the component waves in Fourier analysis. The variation in luminance over space (luminance profile) of the sample shown at the top and enlarged in the first row of the left hand inset can be thought as composed of the addition of the waves shown below and numbered 1–4. The amplitude decreases with their spatial frequency as shown in the right-hand inset.

Fourier analysis is a mathematical technique that decomposes images into components. The components are typically (but not necessarily) sine-wave patterns with a variety of spatial frequencies, orientations and phases. Consider the small sample of the image in Figure 5. An enlarged version of the sample is shown in the left insert and the profile of the luminance over space is shown in the top graph. This waveform can be created by adding together the waveforms numbered 1-4 immediately below. The peak–trough amplitude of these waves is directly proportional to their wavelength. The spatial frequency, f, of the waves is the reciprocal of their wavelength, and so the amplitude is proportional to 1/f. This means that when the logarithm of the amplitude is plotted against the logarithm of the spatial frequency, a straight line with a slope of −1 results. It turns out that most images from nature have a Fourier amplitude spectrum with a slope close to −1 [18].

The visual system has adapted to process natural images. Field [19] argued that the bandwidths of channels tuned to spatial frequency are optimized for a 1/f amplitude spectrum. Their bandwidth remains constant when expressed on an octave scale, so that a similar amount of information is carried by each channel. Atick and Redlich [20] have argued that the shape of the contrast sensitivity function enables images with a 1/f spectrum to be coded efficiently. The contrast sensitivity is low (the channel has low gain) for low spatial frequencies that have a high amplitude, and this conserves metabolic energy. In computational models of the visual system, striped patterns, which are rare in nature and do not conform to a 1/f structure, result in an excess of ‘neural activity’ and a non-sparse distribution of ‘neural’ firing [21]. Wilkins [22] reviewed neuroimaging studies and concluded that images that are uncomfortable to observe are generally associated with an elevated haemodynamic response, consistent with the computation models and suggesting that the discomfort is a homeostatic mechanism that avoids excessive cerebral metabolism.

**Figure 6:**
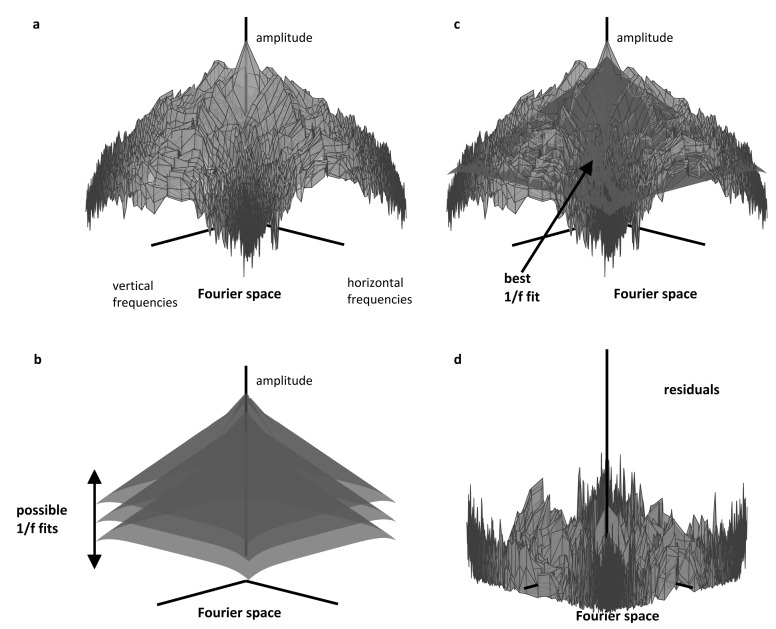
Schematics of the method used in [25]. (**a**) Amplitude spectrum of a natural image in log–log coordinates. Each vertex on the surface has three coordinates: two corresponding to the logarithm of the spatial frequency in the two-dimensional Fourier domain (frequencies with a DC component have been discarded for display purposes). The third (vertical) coordinate corresponds to the amplitude of the spectrum of the image at these frequencies. (**b**) Family of circular regular cones of slope 1/f with a (continuously) varying gain. (**c**) Amplitude spectrum pictured in (**a**) with its best fit amongst the (continuous) family of two-dimensional cones pictured in (**b**). (**d**) Residuals after the cone of best fit is removed. After [25].

It is therefore possible to argue that images are processed inefficiently by the brain if they are unnatural and do not have a 1/f amplitude spectrum. According to this hypothesis, images are uncomfortable to look at when they are processed inefficiently and require excessive metabolism. Observers have been asked to rate the discomfort from images of modern art and of filtered visual noise or shapes [23,24]. For all categories of image, the discomfort was minimal for those images with a 1/f Fourier amplitude spectrum. An algorithm with no free parameters can account for more than 25% of the variance in observers’ ratings of discomfort [25]. The algorithm fitted a cone with a slope of 1/f to the two-dimensional Fourier amplitude spectrum of the grey-level image, and the residuals were weighted by a contrast sensitivity function gleaned from the literature; the size of the residuals predicted discomfort, see Figure 6.

Note that patterns of stripes have a Fourier amplitude spectrum that is not well fit by a cone, consisting of a few component spatial frequencies. They are therefore patterns that produce some of the largest residual scores, and they are uncomfortable to look at.

The algorithm had not hitherto been applied to text, and so in the next study, we assessed its effectiveness in this context. 

## 6. Study 2

Study 2 applied the algorithm to text and showed that it predicted the choice people make when reading text in electronic books. These books, presented on an electronic screen, permit readers to choose the font and the size and to choose from a limited range of colours.

### 6.1. Predicting Readers’ Choice of Text Characteristics

#### 6.1.1. Participants

Seven female and eight male students and staff at the University of Essex, aged 18–63, took part.

#### 6.1.2. Procedure

Each participant was shown publications from iBooks. The books were presented in double-page format on an Apple MacBook Pro with a 15 inch Retina screen. The participants were asked to “make the text as easy to read as possible” using the iBook tools. With the tools, one of eight fonts could be selected (Original, Athelaf, Charter, Georgia, Iowan, Palatino, Servarek and Times New Roman) in a variety of sizes. The text could be black on a white background or on a sepia background, or the text could be white on a black background. When the participants had finished their settings the image of the screen was saved. Each participant adjusted two texts, one from “The Other Woman” by Laura Wilson and one from “The Angel” by Katerina Diamond. The texts were originally presented in their default settings: Original and Times New Roman with an x-height of 2.3 and 2.2 mm, respectively, and line spacing of 6.8 and 4.9 mm. The work was carried out with permission of the University of Essex Ethics committee and in accordance with the Code of Ethics of the World Medical Association (Declaration of Helsinki). Informed consent was obtained.

#### 6.1.3. Results

The final settings of texts had x-heights that ranged from 1.9 to 2.9 mm, with line spacing that ranged from 4.4 to 8.4 mm. There was no consistent change in the x-height or line spacing relative to the original text: respectively, 52% and 63% of settings showed an increase. The ratio of x-height to line spacing ranged from 31% to 46% and also did not change consistently, decreasing in 56% of cases. Although there was no consistent change in typographic parameters, the adjusted text nevertheless showed a consistent change in the residuals calculated by the algorithm: 29/30 settings (14/15 participants) showed a decrease as compared to the original, indicating that the parameters of the text were altered in combination so as to be closer to those of natural images. Seven of the 15 participants changed the background colour from white to sepia and one used a black background for one of the two texts. The residuals were lower for the sepia background because of the reduction in luminance contrast. Nevertheless, for all the eight participants who chose a white background, the residuals were lower for the adjusted text than for the original in every case, averaged for the two samples.

#### 6.1.4. Discussion

In all cases but one, participants adjusted the iBook text in a direction predicted by the final version of the algorithm from [25]. Although the x-height and line separation did not change consistently, the text was adjusted in a manner to which the algorithm was sensitive. The adjustments were such as to make the text more like images from nature, a change consistent with a reduction in discomfort. Given that the algorithm was successful in predicting choice of typographic variables, it was important to ascertain more systematically which typographic variables affected the output of the algorithm. 

### 6.2. Fonts and the Algorithm Output 

#### 6.2.1. Procedure

The passages from Trollope and Eliot were set in the 20 different fonts given in Table 1, 10 pt in size, with a line spacing of 1.15 pt. They were analysed using the final algorithm of [25].

#### 6.2.2. Results

**Figure 7:**
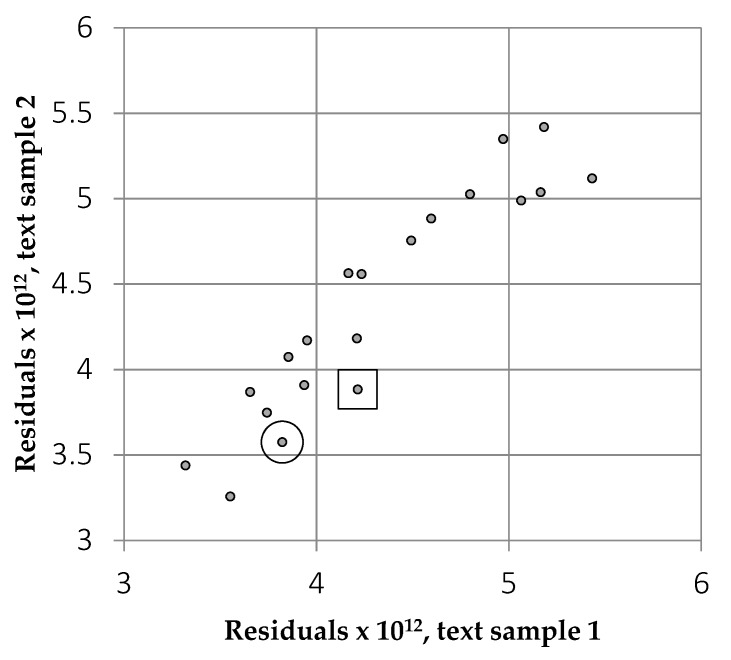
Residuals for the two samples of texts from Eliot and Trollope for each of the 20 fonts listed in Table 1. The lower the residuals, the more similar the text to images from nature. The points for Open Sans and Times are surrounded by a circle and a square respectively.

The residuals obtained for the two text samples are expressed as a scatterplot in Figure 7. The correlation explains 87% of the variance and indicates that the residuals are affected primarily by the font, with little contribution from the textual content. The absolute value of the difference between the text samples averaged 5.0% (SD 2.7%). Table 1 shows the average of the residuals for the two samples. Among the conventional fonts, Open Sans had one of the lowest residuals.

#### 6.2.3. Discussion

The font had a clear effect on the size of the residuals of the images of a page of text. The nominal size of the font was held constant, although there was variation in x-height, which may have contributed to the differences in residuals.

### 6.3. Font Weight

**Figure 8:**
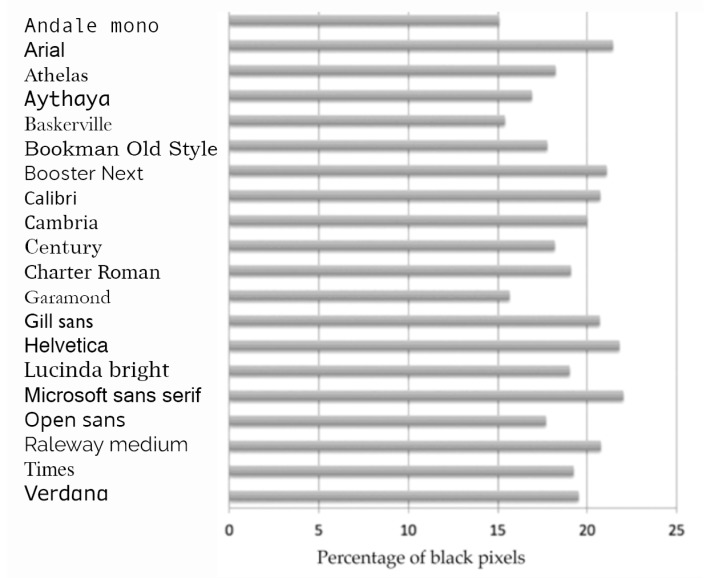
Weights of 20 fonts as estimated from the percentage of black pixels in a line of text.

There were large differences between fonts in the residuals obtained when a 1/f cone was fitted to the two-dimensional Fourier amplitude spectrum of the page using the algorithm [25]. If the page resembles a pattern of horizontal lines, giving large residual scores as a result, then the contrast of that “grating” will be influenced by the *weight* of the font. Weight is typically used to refer to the differences between “light”, “medium” and “bold” versions of fonts but there is continuum of weight that can be assessed from the average thickness of the letter strokes. In order to estimate the weight of the 20 fonts, the sentence “The quick brown fox jumped over the lazy dog” was printed in each of the fonts. The central section of the line from the baseline to the top of those letters without ascenders comprised a rectangular section of the page. The weight was estimated as the number of black pixels divided by the total pixels in the rectangle. The weight for sentences printed in 11, 12 and 14 point were calculated and the correlations between the estimates obtained was high (>0.967). The estimates for 11 point varied 15%–24% as shown in Figure 8.

**Figure 9:**
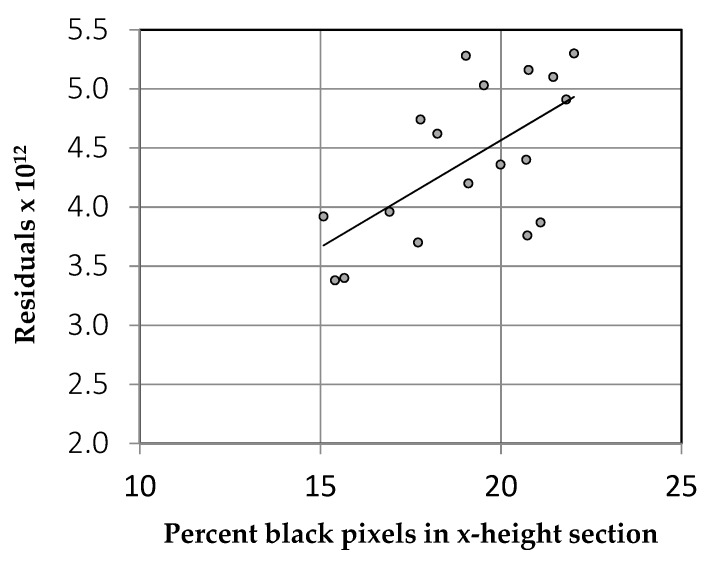
The correlation between font weight and the output of the algorithm (residuals) [25].

There was a moderate but significant correlation (r = 0.624, *p* = 0.003) between the algorithm output and the weight of the font as defined above, shown in Figure 9. The effect of font weight on the algorithm can therefore be understood in terms of the contrast of the striped lines or “grating” formed by the lines of text. 

The next study investigated the effects of the parameters of text layout.

#### 6.3.1. Procedure

Seventy-two samples from Trollope were set in Century, Times, Open Sans and Verdana, 10, 12, and 14 point in size, with 1, 1.15 and 1.5 point interline spacing, with and without justification. They were analysed using the algorithm from [25]. The samples of text with expanded letter spacing were also analysed.

#### 6.3.2. Results

**Figure 10:**
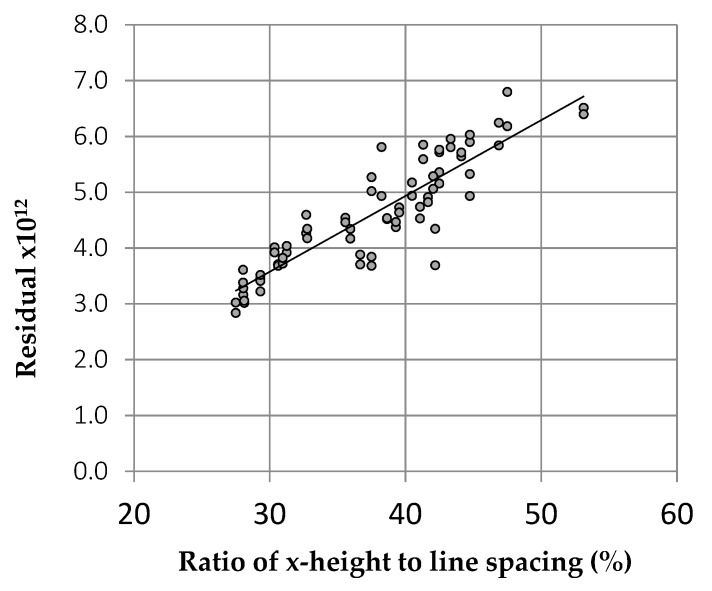
Relationship between the output of the algorithm (residuals) [25] and the ratio of x-height to line spacing for 72 texts, with various fonts, font size and spacing, with and without justification. The font with one of the lowest residuals, Open Sans, had a ratio of x-height to line spacing of 43%, which was large, relative to other fonts. Note that iBooks currently permit the selection of font but *not* the ratio of x-height to line spacing, except in so far as line spacing co-varies with font. The differences in the iBook selections were not therefore attributable to line spacing but to other aspects of the typefaces that the algorithm was also successful at capturing.

There was no relationship between the residuals obtained and the number of words per page (r = 0.03). The residuals were unaffected by the right margin, ragged or justified. The x-height also had little effect. There was a weak tendency for texts with large interline spacing to have lower residuals, but by far the strongest relationship between the output of the algorithm and the typographic variables concerned the ratio of x-height to line spacing. This relationship accounted for 79% of the variance; see Figure 10. Pages in which the text was widely spaced relative to the height of the letters were evidently more similar to images from the natural world, and potentially more comfortable.

The ratio of x-height to line spacing can be likened to the duty cycle of the “grating” formed by horizontal lines of text. The lower the ratio, the greater the departure from a 50% duty cycle and the lower the Fourier power of the “grating” formed by the lines of text. In current typographic practice, the ratio of x-height to line spacing is typically 35%–45%. The present findings suggest that this might usefully be decreased by increasing line spacing. 

## 7. General Discussion

Two mathematical properties of text have been studied: (1) the spatial periodicity of the vertical strokes of letters, which affects reading speed, and (2) the degree of departure from the Fourier amplitude spectrum typical of natural images, which determines visual comfort of images, including text. Overall, the correlation between these two measures for the 20 fonts in Table 1 is only 0.082, suggesting that the measures are independently useful. Open Sans is one of the few fonts that perform well on both measures.

Models of the way in which words are read typically represent (1) the detection and integration of visual features to build letters, and (2) the integration of letter-level information to reach word identification, as described by the original interactive activation model [26] and its descendants [27,28,29]. The approach adopted in this paper differs fundamentally. It treats text as a shape and shows that there are aspects of reading that depend upon the similarity of shapes at various spatial scales, irrespective of their identity as parts of letters or words. 

Many studies have investigated eye movements across text but usually without attention to the detail of font and text design, and often with monocular recording, e.g., [30]. The present findings suggest that detailed binocular recordings that measure vergence will show effects of font and layout on binocular eye movements across text.

The autocorrelation encompasses in a single measure aspects of crowding and spatial uncertainty of letter position, which are key features of recent models of word reading, e.g., [31]. The autocorrelation has a face validity in terms of the cues to vergence that are used following each saccade, and the large effect on reading speed is understandable in these terms, and may even explain why crowding and eccentricity jointly determine reading rate [32]. Nevertheless, it seems likely that crowding and spatial uncertainty are insufficiently captured simply by autocorrelation. The effects of crowding increase with eccentricity, and no account of the decrease in acuity with eccentricity has been taken in the autocorrelation measure used here. This might be significant if the results are to be applied to low vision reading, particularly reading that uses peripheral retina.

There is convergent evidence that in migraine, and perhaps in headache more generally, the visual cortex is hyperexcitable [33], and that as a result, individuals can be particularly susceptible to discomfort from patterns of stripes [14], including stripes in text [16]. In this paper, we have shown how the vertical stripes in text are affected by the selection of font and the horizontal stripes by text layout. It is stripes of this kind and the visual stress [13] they evoke that can have an impact on reading similar to that from low vision. With the advent of electronic text, it is possible without additional cost to choose fonts with little *rhythm* and to increase the spacing between lines of text [34]. It is possible in principle, but not in practice. Currently, the selection of fonts and spacing in electronic books is insufficient to accommodate individual preference or indeed to optimize text according to the principles exposed here; and there are no algorithms available with electronic books to guide users’ choice of font and spacing.

The height of the first peak in the horizontal autocorrelation varies considerably for different fonts, but is high for Sassoon Primary, a font used in schools. Sassoon Primary is read more slowly than Verdana by school children [35], as predicted by the higher autocorrelation of Sassoon Primary, notwithstanding the children’s greater familiarity with this latter font. The question arises as to whether Open Sans might provide a better font for children than those currently in use. There are three aspects of the font that would then need to be changed, however. The q would need a tail to distinguish it from p. Similarly, the b and d would need to be distinguished, perhaps by removing the base of the stem of the b or adding a small tail to the stem of the d. Finally the upper case i and lower case L would need to be distinguished, for example by adding a small tail at the base of the lower case L. (Interestingly, these are changes that were systematically applied in a study that created a new font, Eido, to improve low-vision reading [36].) The changes could be very slight and would in any event have little or no effect on the autocorrelation or residuals. The alterations would be simple to make and would be admissible within the terms of the open license.

## 8. Conclusions

The general principles and methods described in this paper can be applied to all languages and all orthographies and can be used to guide the design of any typeface and any written material. For example, spatial periodicity has been shown to affect the reading of Chinese logograms [37]. The present findings suggest, surprisingly, that font design has the potential to affect reading fluency and comfort appreciably. Similar algorithms may be applicable to music manuscripts [38].

## Figures and Tables

**Table 1:** A total of 20 fonts showing the mean of the first peak in the horizontal autocorrelation (AC peak) and the residuals after fitting a 1/f cone to the Fourier amplitude spectrum.